# Tailoring of arteriovenous graft-to-vein anastomosis angle to attenuate pathological flow fields

**DOI:** 10.1038/s41598-021-90813-3

**Published:** 2021-06-09

**Authors:** Dillon Williams, Eric C. Leuthardt, Guy M. Genin, Mohamed Zayed

**Affiliations:** 1 Vascular Surgery Biomedical Research Laboratory, Washington University School of Medicine, Saint Louis, MO 60613 USA; 2 Center for Innovation in Neuroscience and Technology, Department of Neurological Surgery, Washington University School of Medicine, Saint Louis, MO 60613 USA; 3 Department of Biomedical Engineering, Washington University, Saint Louis, MO 63130 USA; 4 NSF Science and Technology Center for Engineering MechanoBiology, Washington University in St. Louis, Saint Louis, USA

**Keywords:** Computational biology and bioinformatics, Renal replacement therapy

## Abstract

Arteriovenous grafts are routinely placed to facilitate hemodialysis in patients with end stage renal disease. These grafts are conduits between higher pressure arteries and lower pressure veins. The connection on the vein end of the graft, known as the graft-to-vein anastomosis, fails frequently and chronically due to high rates of stenosis and thrombosis. These failures are widely believed to be associated with pathologically high and low flow shear strain rates at the graft-to-vein anastomosis. We hypothesized that consistent with pipe flow dynamics and prior work exploring vein-to-artery anastomosis angles in arteriovenous fistulas, altering the graft-to-vein anastomosis angle can reduce the incidence of pathological shear rate fields. We tested this via computational fluid dynamic simulations of idealized arteriovenous grafts, using the Bird-Carreau constitutive law for blood. We observed that low graft-to-vein anastomosis angles ($<20^{\circ }$) led to increased incidence of pathologically low shear rates, and that high graft-to-vein anastomosis angles ($>40^{\circ }$) led to increased incidence of pathologically high shear rates. Optimizations predicted that an intermediate  ($\sim 30^\circ$) graft-to-anastomosis angle was optimal. Our study demonstrates that graft-to-vein anastomosis angles can significantly impact pathological flow fields, and can be optimized to substantially improve arteriovenous graft patency rates.

## Introduction

Hemodialysis is provided to more than 300,000 Americans annually who have end stage renal disease^1^. This life-sustaining procedure facilitates removal of circulating metabolic toxins in a patient’s blood stream by diverting arterial flow into a hemodialyzer. The blood is then returned in real-time into the venous circulation^1^. Arteriovenous grafts are commonly implanted in patients who require hemodialysis to provide direct access to a patient’s arterial and venous blood streams^2^. Non-autologous polytetrafluoroethylene (PTFE) arteriovenous conduits are most common, and are anastomosed to an artery at one end and to a vein at the other end. The body of the graft is then tunneled subcutaneously to facilitate percutaneous hemodialysis cannulation in the future^3, 4^.

**Figure 1:**
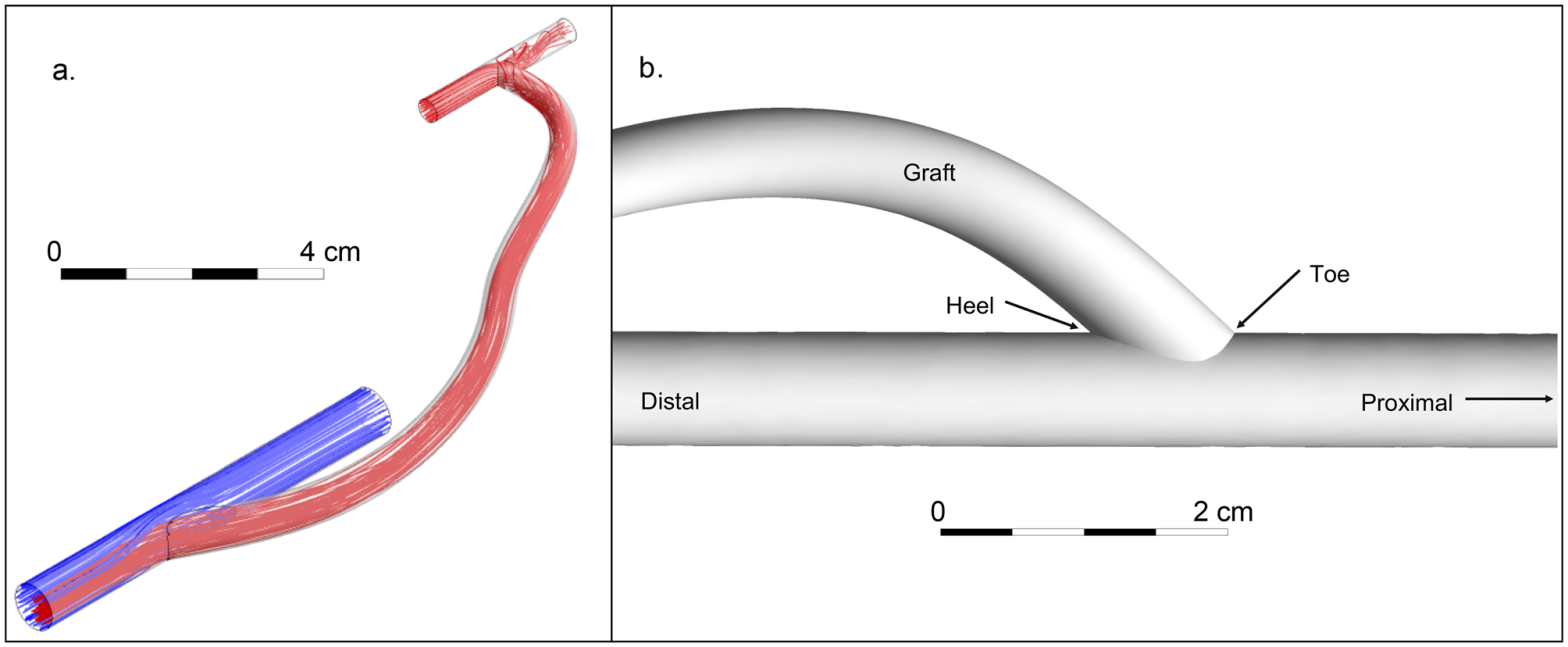
(**a**) Idealized model of a brachio-axillary arteriovenous graft in an adult human. For illustration, streamlines estimated by computational fluid dynamics analysis are shown: red streamlines represent arterial blood flow, and blue streamlines represent venous blood flow. (**b**) Venous end of the graft demonstrating the beveled hood of the arteriovenous graft-to-vein anastomosis.

One of the major configurations for arteriovenous graft surgical implantation is in the upper arm between the brachial artery (near the elbow) and the axillary vein (near the arm pit)^3^. Accordingly, brachio-axillary arteriovenous grafts (Fig. 1a) are tunneled in the upper arm to facilitate routine hemodialysis cannulation while the patient is sitting comfortably. The arterial inflow through the brachial artery typically ranges 3 to 5 mm in diameter with a maximum blood flow velocity ranging between 60 cm/s and 100 cm/s^5,6^. The venous outflow through the axillary vein typically ranges 6 to 10 mm in diameter, and has a mean blood flow velocity of approximately 15 cm/s^7^. To limit arterial steal (diverting too much arterial blood into the arteriovenous graft), tapered brachio-axillary arteriovenous graft conduits are commonly used in adult patients in clinical practice^8,9^. These grafts are typically tapered from 4 mm at the graft-to-artery anastomosis to at least 7 mm at the graft-to-venous anastomosis (Fig. 1b)^10^. The angle of the graft-to-vein anastomosis is typically chosen empirically by the surgeon at the time of implantation, and is mostly based upon geometric intraoperative constraints during graft surgical implantation^11^.

Despite advances in arteriovenous graft material technology, brachio-axillary grafts failure rates can be as high as 30% within the first 12 months^4,12^. Arteriovenous graft thrombosis can be problematic if graft patency is not immediately restored, or alternative arteriovenous access placed to facilitate continued hemodialysis. This leads to a high degree of repeated interventions and hemodialysis access placement, which leads to increased overall disease morbidity^13^. As many as 80% of arteriovenous grafts develop high grade stenoses at the graft-to-vein thrombosis leading to eventual graft thrombosis and loss of hemodialysis access^4,14^. It is postulated that stenosis at the graft-to-vein anastomosis develops due to pathological flow fields that cause intimal damage^15^, leading to myointimal hyperplasia^16^, and eventual activation of circulating clotting factors^17–21^. Shear strain rates below the threshold of 50 s$^{-1}$ show increased fibrin deposition that can stimulate coagulation^18,22^. On the other hand, shear rates elevated above 1000 s$^{-1}$ can also lead to micro injuries in the vessel wall and lead to platelet aggregation^22,23^. It is therefore recognized that flow rates within a physiological range are necessary to minimize risk of thrombosis at the graft-to-vein anastomosis and sustain graft patency.

Wall shear stresses at a junction between two connected pipes is strongly impacted by the attachment angle^24^. In the vasculature, this is especially true, as demonstrated in arteriovenous fistulas (veins that are directly anastomosed to arteries as an autogenous alternative to hemodialysis access)^25^. In fistulas, the vein-to-artery anastomosis angle can greatly impact pathological flow fields in the vicinity of the arteriovenous anastomosis^26–28^. However, a similar dynamic flow analysis of the problematic graft-to-vein anastomosis in arteriovenous grafts has yet to be conducted. We hypothesized that identifying an optimal range for graft-to-vein anasotmosis angles can potentially decrease the risk of pathological flow fields in this segment of interest, and test this using a series of computational dynamics simulations. Results from our study may ultimately improve arteriovenous graft surgical implantation techniques, and help decrease morbidity associated with hemodialysis access.

## Methods

We estimated the pulsatile flow fields of blood in a brachio-axillary arteriovenous graft system with a graft in place through pressure-based transient computational fluid dynamics (CFD) simulations. An estimate of the Reynolds number, $\mathrm {Re}=\rho u D_H/\mu$ prompted us to use a viscous, laminar model. Taking blood density $\rho$ = 1060 kg/m$^3$, blood dynamic viscosity of *μ* = 0.0035 Pa$\cdot$s, characteristic length scale $D_H$ as the arterial diameter of 4 mm, and maximum velocity of  *u* = 1.5 m/s yielded an estimate of $\mathrm {Re}\approx 1800$, which is within the laminar flow range.

### Physical model and computational mesh

A series of idealized, straight arteriovenous grafts were constructed in commercial CAD software (DesignModeler, ANSYS, Canonsburg, PA) based upon the aforementioned data for typical arterial and venous presentations. In each case, the inner diameter of the artery was 4 mm, and the inner diameter of the vein was 8 mm. The arteriovenous graft was tapered from a 4 mm diameter on the arterial end to a 7 mm diameter on the venous side. These dimensions were chosen to fit both within the range of 3–5 mm for the brachial artery, and within the range of 6–10 mm for the axillary vein. The length of the arteriovenous graft was 150 mm ± 2 mm, depending on the arteriovenous graft-to-vein anastomosis angle. To assess the range of venous anastomosis configurations that may be utilized by a surgeon at the time of implantation, we studied the range of anastomosis major axis sizes that are used in our clinical practice, ranging from a circular anastomosis with a diameter of 7 mm, corresponding to a 90° anastomosis angle, down to an elliptical anastomosis with a major axis of 30 mm, corresponding to an anastomosis angle of 13°. The major axis was defined as the maximum length of a line bisecting the ellipse formed when the cylindrical, conical graft intersected the cylindrical vein. The 7 mm, 8 mm, 10 mm, 15 mm, 25 mm, and 30 mm anastomosis major axis lengths corresponded to graft-to-vein anastomosis angles of 90°, 60°, 45°, 30°, 15°, and 13°, respectively.

For all simulations, the vein length was 72 mm, with the length distal to the graft held at 25 mm and that proximal to the graft ranging from 40 mm to 17 mm depending on the anastomosis angle. This was done to ensure that the proximal and distal sections of the vein were sufficiently long for flow fields to be fully developed before and after the anastomosis, so that regions over which pathological flow rates were observed were fully contained within the model.

**Figure 2:**
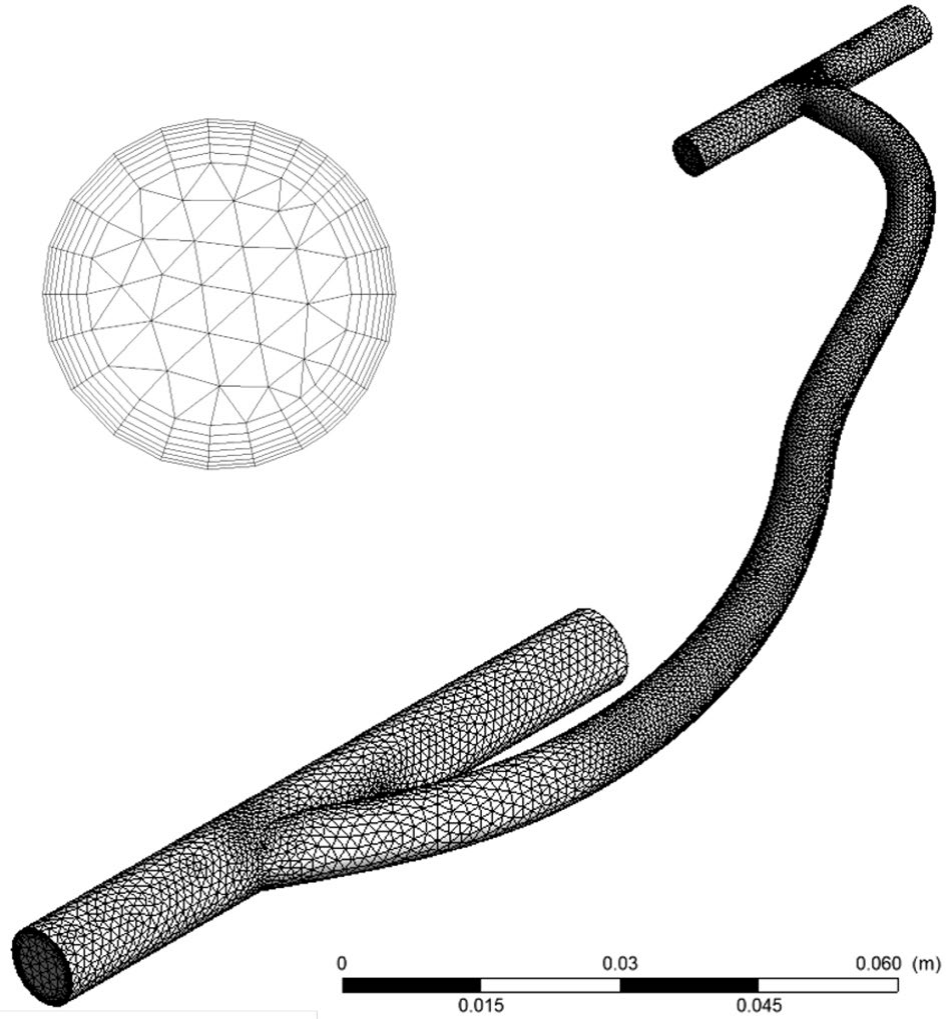
The mesh of the idealized arteriovenous model. The smaller diameter cylinder represents the artery, and the larger diameter cylinder represents the vein. The two were connected by an arteriovenous graft. Shown is a model with a 30° graft-to-vein anastomosis angle. The inset is a close up of the venous outlet that provides a representation of the mesh refinement at the boundary layer. The maximum aspect ratio of the boundary layer elements was 10:1. In all simulations, the vein length was 72 mm, with the length distal to the graft held at 25 mm and the length proximal to the graft ranging from 40 mm to 17 mm depending on the anastomosis angle.

A computational mesh on which to estimate flow fields was generated from this model using ANSYS Fluent (ANSYS, Canonsburg, PA). Care was taken to ensure that discretization was not distorted in regions of high curvature and at the edges of the anastomosis. For each simulation, mesh size was refined until convergence was reached. Meshes were refined so as to ensure satisfactory levels of element skewness and orthogonality^29^. The Blasius solution (Eq. 1) was used as a first order approximation of the maximum height of the boundary layer to aid in meshing the boundary layer^30^:

1 $$\begin{aligned} \delta \approx 4.91x/{\sqrt{{\mathrm {Re}}_{x}}} \end{aligned}$$

where $\delta$ is the boundary layer thickness, *x* is the distance along the plate, and the $Re_x$ is the Reynolds number. Convergence for the standard anastomosis models required between 200, 000 and 250, 000 elements (Fig. 2). An inflation mesh was set at the graft wall to ensure complexities at the boundary layer could be resolved. Because the intersection between the graft and the vein was neither the flat plate geometry of the Blasius solution nor the straight tube configuration for its cylindrical equivalent, this was only an approximation, this estimate was used only as an initial approximation, and results were checked to ensure that the boundary layer did not extend beyond the highly refined region. The inflation created a 1 mm area of seven prism elements expanding at a rate of 1.2 at the walls of the artery, vein, and arteriovenous graft (Fig. 2, circular inset). In all cases, the boundary layer size estimated via the Blasius solution proved adequate for this purpose. The maximum aspect ratio of any element was 10:1.

### Modeling of blood

Blood was modeled as a non-Newtonian fluid following the Navier-Stokes equation and the incompressible mass continuity equation:

2 $$\begin{aligned}&\frac{\partial }{\partial t} (\rho u_{i}) + \frac{\partial }{\partial x_{j}} (\rho u_{i} u_{j} + p \delta _{ij} - \tau _{ji} ) = 0 \end{aligned}$$

3 $$\begin{aligned}&\frac{\partial \rho }{\partial t} + \frac{\partial }{\partial x_{i}} (\rho u_{i}) = \frac{\partial u_{i}}{\partial x_{i}} = 0 \end{aligned}$$

in which Einstein notation was used so that $u_i$ is the velocity vector, $\tau _{ji}$ is the stress tensor, Kronecker’s delta $\delta _{ij}$ functions as an identity tensor, and repeated indices imply summation. *p* is the hydrostatic pressure. Because the flow was incompressible, Eq. (4) was identically satisfied. The Bird-Carreau constitutive law was used to represent the non-Newtonian, pseudo-plastic nature of blood, as is appropriate for oscillatory flow with high Womersley number ($\alpha =R\sqrt{\omega \rho /\mu }$, in which $\omega$ represents heart rate, in radians per second, and *R* is the artery radius)^31–33^:

4 $$\begin{aligned} \tau _{{ij}}=2\dot{\gamma }_{{ij}}\mu _{{eff} }(\mathrm {De}) \end{aligned}$$

in which the strain rate tensor $\dot{\gamma }_{{ij}}$ is:

5 $$\begin{aligned} \dot{\gamma }_{{ij}}=\frac{1}{2} \left( {\frac{\partial u_{i}}{\partial x_{j}}}+{\frac{\partial u_{j}}{\partial x_{i}}} \right) \end{aligned}$$

and effective viscosity changes with the Deborah number, $\mathrm {De}$:

6 $$\begin{aligned} {\displaystyle \mu _{{eff} }(\mathrm {De})=\mu _{{\inf } }+(\mu _{0}-\mu _{{\inf } })\left( 1+\left( \mathrm {De}\right) ^{2}\right) ^{\frac{n-1}{2}}} \end{aligned}$$

where *n* is a constitutive parameter and $\text{De}= \lambda{\dot{\gamma}}$. Here, $\lambda$ is the characteristic relaxation time of the blood, and the effective shear strain rate is $\dot{\gamma }=\sqrt{2I_2}$^32,34^, in which $I_2$ is the second invariant of $\dot{\gamma }_{{ij}}$. The parameters used are listed in Table 1.

**Table 1:** Material properties of blood^35^.

Density, $\rho$ (kg/m$^3$)	1060
Viscosity at zero shear rate, $\mu _{0}$ Pa s	0.0056
Viscosity at infinite shear rate, $\mu _{{\inf }}$ Pa s	0.0035
Relaxation time, $\lambda$ (s)	3.313
Power index, *n*	0.3568

### Boundary conditions

**Figure 3:**
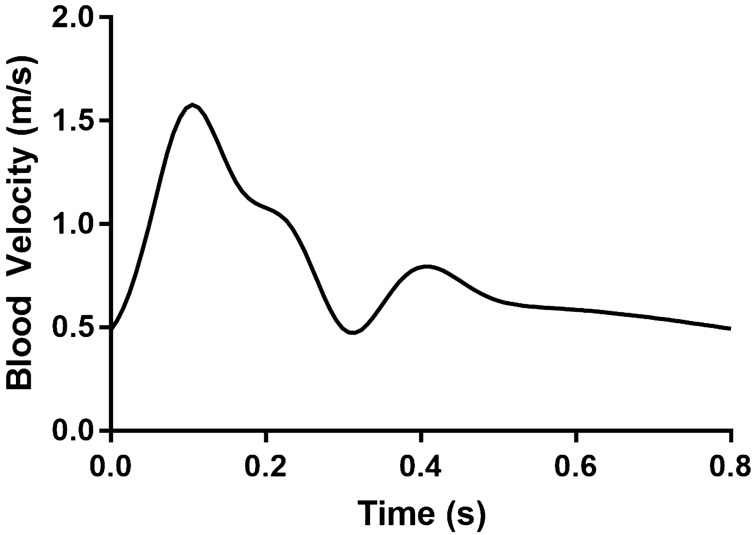
Velocity function for the arterial inlet.

The inlet condition for the artery was uniform flow defined by a centerline velocity function. This velocity waveform was based off ultrasound data from the centerline velocity measured in the brachial artery of patients with dialysis access^5,36–38^, and was reconstructed using Gaussian wave fitting^39^ converted to a Fourier series:

7 $$\begin{aligned} f(t)={a_0}+\sum _{n=1}^\infty ~a_n \cos \left( {\omega n t}\right) +\sum _{n=1}^\infty ~b_n \sin \left( {\omega n t}\right) \end{aligned}$$

where $a_n$ and $b_n$ are curve fit parameters, *t* is the simulation time, and $\omega$ is angular frequency. Adjusting this allowed effective control of simulated heart rate. Fitting was done in MATLAB (The Mathworks, Natick, MA) using eight terms. The coefficients and frequency can be found in the Appendix. Figure 3 shows the resulting function that was used as the arterial inlet velocity. The regions of interest for this study were sufficiently downstream from the inlet to ensure a fully developed profile. Specifically upstream of the arteriovenous anastomosis the velocity profile followed a Womersley profile, as is appropriate for cases of high Womersley. For $\alpha$ greater than 2, inertial forces dominate over viscous forces; in peripheral veins and arteries the Womersley number is between 2 and 3^40^. The volumetric flow rate into the arteriovenous graft was measured to be 700 ml/min, which is within the range of healthy flow rate for an arteriovenous graft^41^. The venous system does not experience significant pulsatile flow^42^. The flow from the distal side of the vein to the proximal side of the vein was specified as a constant, centerline velocity of 15 cm/s, as appropriate for flow in the axillary vein^43^.

Outlet boundary conditions are a well known challenge for vascular flow simulation. Although standard practice is for pipe flow to use pressure outlet conditions^29^, this fails to account for the downstream effects that are present in the circulatory system. A common approach is therefore to use an outflow boundary condition for simulating vascular systems, especially for arteriovenous grafts^44,45^. Following National Kidney Foundation KDOQI Clinical Practices^41^, inflow was distributed so that 90% was sent to the graft and 10% to the distal artery. An outflow boundary condition was also imposed at the proximal end of the vein. No-slip boundary conditions were used along the vessel walls, and vessels were treated as rigid.

### Solution procedure

Equations were solved using the ANSYS Fluent CFD solver (ANSYS, Canonsburg, PA). The semi-implicit method for pressure-linked equations (“SIMPLE”) pressure-velocity coupling method was used with second order spatial discretization and first order transient discretization. The pressure-implicit with splitting-operations (“PISO”) method was also used, with second order spatial discretization and transient discretization, with negligible differences in results but with increased computation time.

Hybrid initialization was done by solving Laplace’s equation, $\nabla ^{2}\varphi =0$, in which $\varphi$ is a potential function defining the velocity field: $\mathbf {u} = \nabla \varphi$. Boundary conditions used in the hybrid initialization for the walls, inlets, and outlets of the system were:

8 $$\begin{aligned}&\left. \frac{\partial \varphi }{\partial n} \right| _{wall} = 0 \end{aligned}$$

9 $$\begin{aligned}&\left. \frac{\partial \varphi }{\partial n} \right| _{inlet} = u_{\bot } \end{aligned}$$

10 $$\begin{aligned}&\varphi _{outlet}= 0 \end{aligned}$$

For the transient calculation, convergence was achieved with a time step of 0.0025 s for 320 time steps, which enabled simulation of one pulse with high accuracy. 20 iterations or fewer were required at each time step, as is common practice^29^. Convergence criteria for the continuity, *x*-velocity, *y*-velocity, and *z*-velocity were all set at an absolute tolerance of 0.001.

### Quantification

We defined metrics to quantify the extent and magnitude of pathological shear stresses experienced by the vein walls during a single cardiac cycle. For low shear strain rates, we defined a metric $\Gamma _{low}$ as the integral of the ratio of area of the vein wall experiencing pathologically low shear strain rates divided by the shear rate over those areas:

11 $$\begin{aligned} {\Gamma _{low}} = \frac{\dot{\gamma }_{min}}{A_0}\int _{S_{low}} \frac{1}{\dot{\gamma }}dA \end{aligned}$$

where the normalization factor $\dot{\gamma }_{min}$ = 1 s$^{-1}$, $A_0$ is the entire area of the model, and $S_{low}$ represents the regions of the vein walls over which shear strains rates of $\dot{\gamma }\le 50$ s$^{-1}$ were predicted.

For shear high strain rates we defined $\Gamma _{high}$ as the integral of the ratio of area of the vein wall experiencing pathologically low shear strain rates multiplied by the shear rate over those areas:

12 $$\begin{aligned} {\Gamma _{high}} = \frac{A_0}{\dot{\gamma }_{max}}\int _{S_{high}} {\dot{\gamma }}dA \end{aligned}$$

where the normalization factor is $\dot{\gamma }_{max}$=3000 s$^{-1}$, and $S_{high}$ is the regions of the vein walls over which by the shear strain rates of $\dot{\gamma }\ge 1000$ s$^{-1}$ were predicted. Note that the length of the vein both proximal and distal to the anastamosis were set in all simulations to be sufficiently long to ensure fully developed flow fields both before and after the graft. While having too short of a vein could affect the measures of flow pathology, having too long of a vein did not, as the region over which pathological flow fields occurred was not expanded by extension of the vein beyond a critical length.

## Results

**Figure 4:**
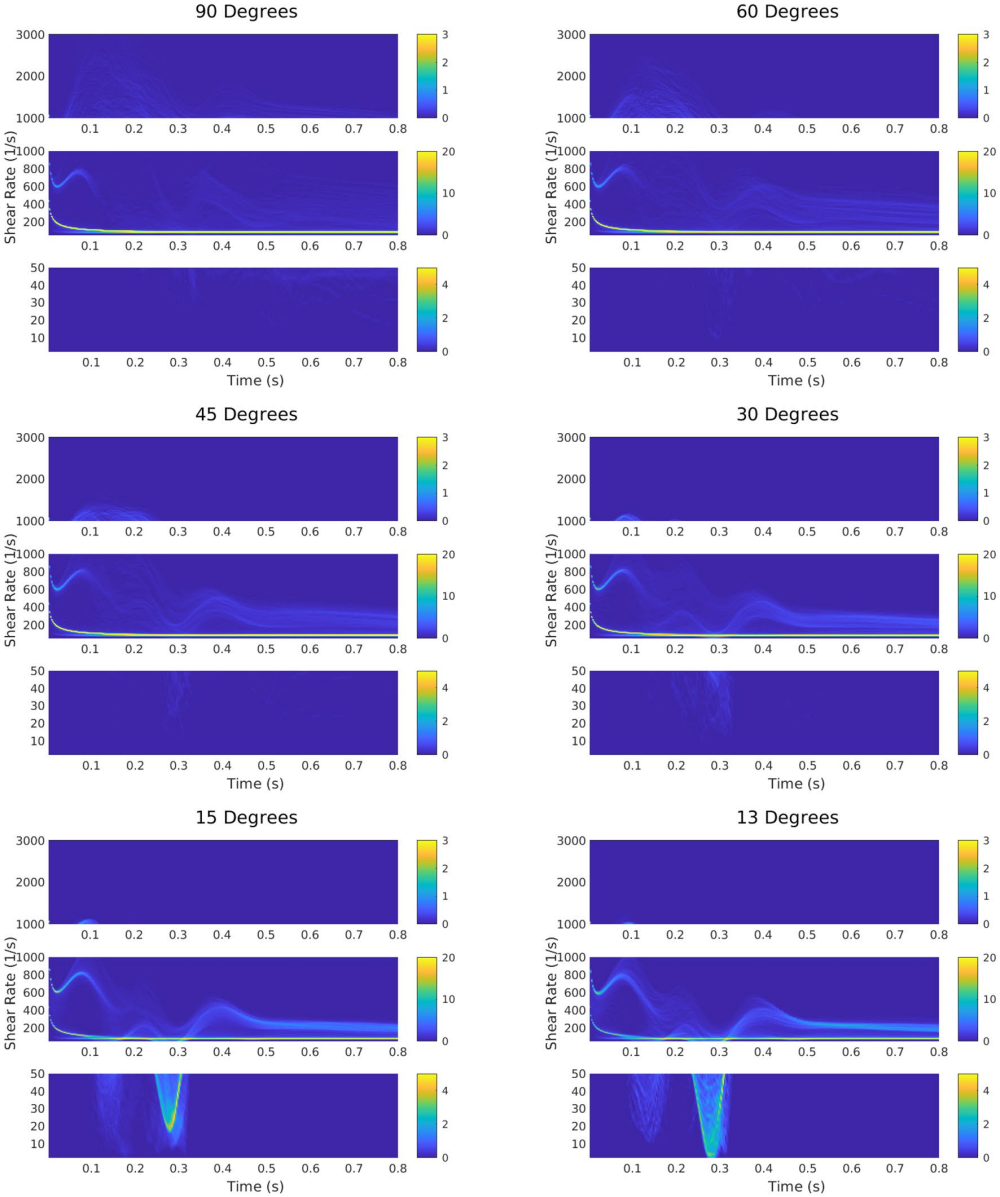
Maps of the percentage of the vein wall experiencing physiological and pathological shear rates over the course of one heart beat, for a range of anastomosis angles. The maps are separated into three sections: pathologically low wall shear rates ($0-50\:s^{-1}$), physiological wall shear rate ($50-1000\:s^{-1}$), and pathologically high wall shear rate ($1000-3000\:s^{-1}$). Contours represent shear rates over the entire wall area, both distal and proximal to the anastomosis.

Blood flow entering the axillary vein from a brachio-axillary arteriovenous graft led to perturbations in the laminar venous blood outflow (Fig. 4). To quantify the degree to which blood flow was pathologically perturbed over one heart cycle, the shear rate was recorded at each of the mesh cells on the vein wall for each time point. This is displayed on the color maps that show the distribution of shear rate during one heart beat (Fig. 4). These distributions revealed an important role for the graft-to-vein anastomosis angle in determining the fraction of the vein wall that experienced pathological shear rates.

**Figure 5:**
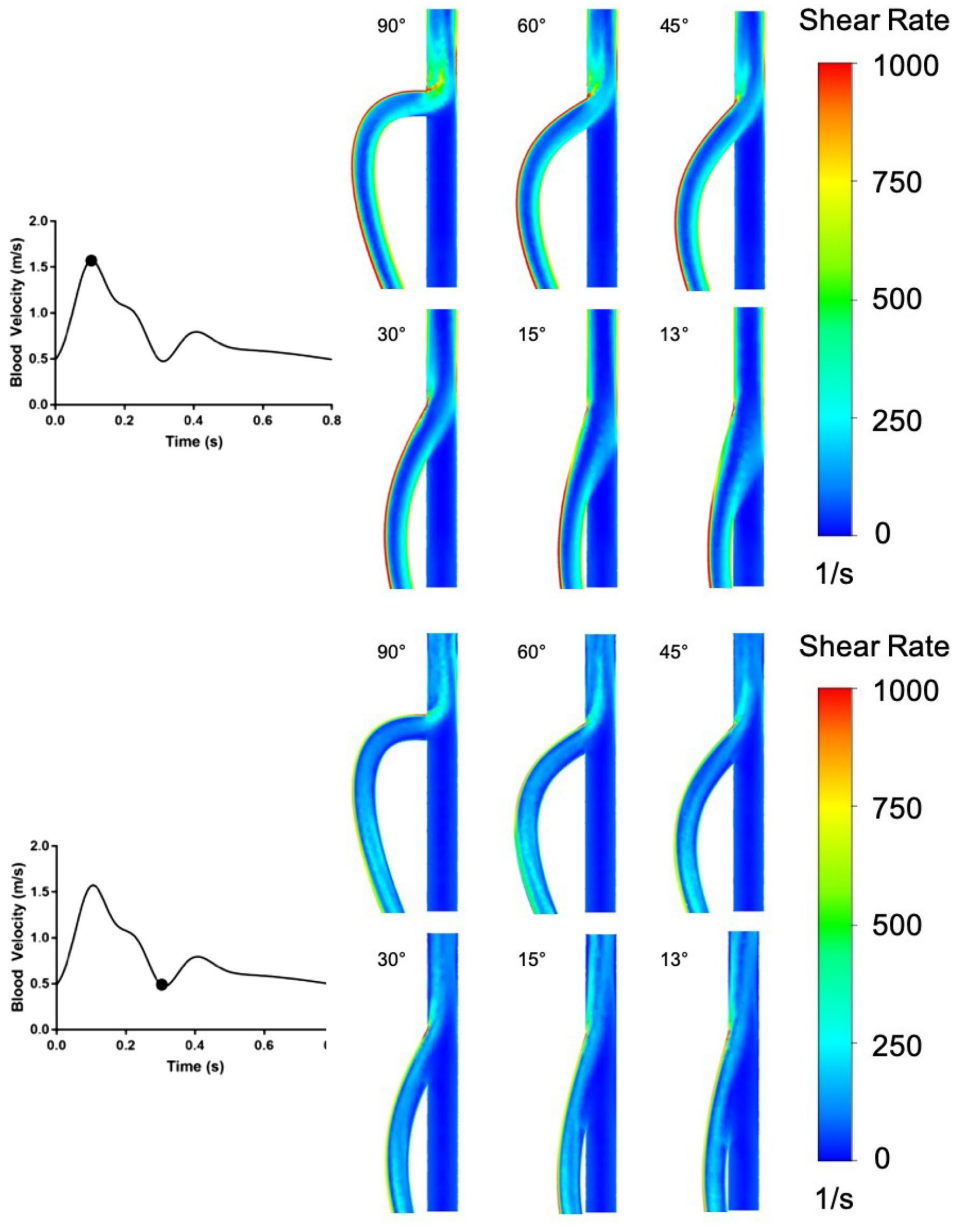
Cross section of anastomoses at selected times over the course of a heartbeat. Top panel: 0.1 s through the heart beat, which was shown to be a time point of excessive high shear rate for all graft-to-vein anastomosis angles considered. Bottom panel: 0.3 s through the heartbeat, which was shown to be a time point of excessive low shear rate for all graft-to-vein anastomosis angles considered. Contours represent shear rates over the entire wall area, both distal and proximal to the anastomosis.

Color maps demonstrated that the graft-to-vein anastomosis angle clearly impacted the shear environment of the adjacent vein wall. Additionally, the data demonstrated the extent of how blood entering the vein from the graft was affected by the graft-to-vein anastomosis angle (Fig. 4). For a 90$^{\circ }$ anastomosis angle, most of the pathological flow fields were in the high shear rate range early in the heart cycle. Conversely, for a 13$^{\circ }$ graft-to-vein anastomosis angle, very few regions of high shear rates were evident in the early stages of the heart cycle, but pathologically low shear rates were evident midway through the heart cycle. Therefore, the data demonstrate that the graft-to-vein anastomosis angle can clearly impact pathological shear rates at different time intervals in the heart cycle. Note that Figs. 4 and 5 show shear rates over the entire vein wall area, both distal and proximal to the anastomosis.

To further understand how the shear environment was affected by the graft-to-vein anastomosis angle, we plotted flow fields over cross-sections of the anastomoses at the peak of high wall shear rate (0.1 s into the 0.3 s heart beat shown) and at the peak of low wall shear rate (0.1 s into the 0.3 s heart beat shown, Fig. 5). At the peak of high shear rate, 0.1 s into the heart beat, high wall shear rate localized to the distal end of the anastomosis and to the opposite vein wall (displayed in red in Fig. 5). The incidence of this high shear rate decreased with decreasing the graft-to-vein anastomosis angle. At the peak of low shear rate, 0.3 s into the heart beat, low wall shear rates localized on the vein wall just distal to the anastomosis area and opposite to the side of the anastomosis. Therefore, high and low shear rates can impact different anatomical regions along the graft-to-vein anastomosis.

**Figure 6:**
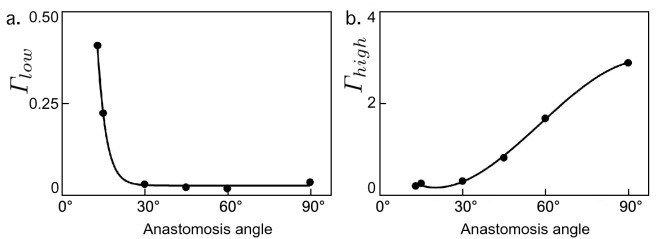
Summary metrics showing the degree to which differing anastomosis designs induced (**a**) pathologically low shear strain rates over the vein wall over the course of a single cardiac cycle, and (**b**) pathologically high shear strain rates over the vein wall over the course of a single cardiac cycle. These summary metrics suggested that the fraction of vein wall area experiencing pathologically high shear strain rates diminished with decreasing graft-to-vein anastomosis angle, while that experiencing pathologically low shear strain rates was low for anastomosis angles above 30$^\circ$.

These summary metrics suggested that the fraction of vein wall area undergoing pathologically high shear strain rates diminished with decreasing the graft-to-vein anastomosis angle, while that undergoing pathologically low shear rates stay low from 90$^\circ$ to 30$^\circ$ then increase rapidly (Fig. 6).

These data suggest that a 30$^\circ$ graft-to-vein anastomosis angle would present the healthiest shear rate environment of the options studied. Increasing the angle of attachment past 30$^\circ$ leads to a rise in area of the vein wall experience unhealthy high shear rate, and decreasing the angle below 30$^\circ$ leads to a rise in area of the vein wall experiencing unhealthy low shear rate. Our data demonstrates that the graft-to-vein anastomosis angle of 30$^\circ$ provided optimal flow fields.

## Discussion

Here we demonstrate that the graft-to-vein anastomosis of a brachio-axillary arteriovenous graft can develop severe pathological flow fields. It is understood that these flow fields are a major contributor to myointimal hyperplasia at the graft-to-vein anastomosis that ultimately lead to stenosis, graft failure, and thrombosis^16,18,20,46^. In this study we observed that low graft-to-vein anastomosis angles that are $<20^{\circ }$ caused a substantial increase of pathologically low shear rates. On the other hand, high graft-to-vein anastomosis angles that are $>40^{\circ }$ caused an increase of pathologically high shear rates. A graft-to-vein anastomosis angle of 30$^\circ$ provided the most optimal flow fields with minimal incidence of pathological shear rates. Our study findings provide important insights that can impact arteriovenous graft implantation techniques, and potentially decrease graft failure rates and morbidity in patients who receive brachio-axillary graft implants to facilitate hemodialysis^4^. Future research will explore a the range of optimal graft-to-vein anastomosis angles relative to different diameter axillary vein diameters.

For patients who require chronic hemodialysis, placement of an arteriovenous graft can be a life-saving alternative to an arteriovenous fistula^47^. Although fistulas are usually the preferred primary method to facilitate hemodialysis access, there are many patients who do not have suitable superficial venous anatomy for fistula placement^47,48^. Accordingly, in an aging population with a rising incidence of end stage renal disease, the use of arteriovenous grafts is anticipated to continue to increase over the coming years^4^. However, the 1 year primary patency of arteriovenous grafts varies between 40-57%, and the 1 year secondary patency is between 62 and 78%^4,48^. Clearly, technical challenges persist in maintaining adequate arteriovenous graft patency, and this is a clear impetus for better characterization of the underlying complicating factors^16,22^.

The graft-to-vein anastomosis angle at which arteriovenous grafts are surgically implanted are entirely up to the discretion of the operating surgeon^4^. Depending on the techniques used to subcutaneously tunnel the arteriovenous graft, the patient’s body habitus, and the axillary vein diameter, the graft-to-vein implantation angle can vary significantly from one patient to another. Various studies have explored ways to decrease graft associated thrombotic complications. For example, adjunct therapy with antiplatelet and anticoagulant therapy has been used as means to reduce graft thrombosis, but several studies have that does not provide any clinically meaningful benefits and may actually increase the risk of bleeding^49–51^. Other studies have attempted modifications to the graft-to-vein anastomosis to reduce the risk of myointimal hyperplasia and graft thrombosis. For example, a study incorporated a venous cuff at the graft-to-vein anastomosis segment, but this graft modification demonstrated no significant impact on graft patency^52^. Our study findings suggest that perhaps a fixed and optimized graft-to-vein anastomosis angle may be a novel solution to this ongoing issue. We envision these grafts would be manufactured at pre-set fixed optimized angles, and would be selected and implanted in patients relative to their native axillary vein diameters as a strategy to improve graft primary and secondary patency rates.

We acknowledge there are some limitations associated with our study. While it is a first step in understanding the behavior this fluid system, our simulations were not able to entirely account for all physiological variables that may impact arteriovenous graft flow. Some limitations of this study are that blood flow parameters were held within the physiological range for all simulations, and vein diameters were not varied. Hypertension is a common co-morbidity of diabetes^53^. Left unregulated, hypertension would be a consideration for the optimization of anastomosis angle as higher or lower flow rates could shift Fig. 6b. However, coexisting diabetes and hypertension is typically managed pharmacologically, with antihypertensives such angiotensin-converting enzyme inhibitors, angiotensin receptor blockers, or thiazide diuretics^53^. Although the size of the vasculature relevant to arteriovenous grafts can vary from patient to patient, the pathologically high and low flow fields were predicted to be localized in all cases to the anastomosis. Because these fields are dominated by the local geometry of the anastomosis rather than by geometry of the vessels, the absolute sizes of the vessels would not be expected to affect the optimization. Lastly, our simulations included blood vessel models that are static. Improving the model to take into account dynamic behaviors of peripheral arteries and veins would further refine our future study findings.

## Conclusion

The graft-to-vein anastomosis angle of brachio-axilllary arteriovenous grafts affects pathological flow fields that arise within the adjacent vein. Optimizing the graft-to-vein anastomosis angle can enhance these flow fields, reduce the incidence of pathological shear strain rates, and may ultimately improve graft patency rates among hemodialysis patients.

## Supplementary Information

Supplementary Information.
