# Supplementary material for: Tailoring of arteriovenous graft-to-vein anastomosis angle to attenuate pathological flow fields
Source: Sci Rep. 2021 Jun 9;11:12153. doi: 10.1038/s41598-021-90813-3 (PMC8190231; doi:10.1038/s41598-021-90813-3)
Supplement: Supplementary file 1 — Supplementary Information. [file 41598_2021_90813_MOESM1_ESM.pdf]

Supplemental Material: Tailoring of Arteriovenous Graft-to-Vein Anastomosis Angle to Attenuate Pathological Flow Fields

Dillon Williams[1,2], Eric C. Leuthardt[2,3], Guy M. Genin[2,3,4,*], and Mohamed Zayed[1,2,3,*]

[1]Vascular Surgery Biomedical Research Laboratory, Washington University School of Medicine, Saint Louis, MO 60613
[2]Center for Innovation in Neuroscience and Technology, Department of Neurological Surgery, Washington University School of Medicine, Saint Louis, MO 60613
[3]Department of Biomedical Engineering, Washington University, Saint Louis, MO 63130
[4]NSF Science and Technology Center for Engineering MechanoBiology, Washington University in St. Louis
[*]genin@wustl.edu, zayedm@wustl.edu

Supplemental material

The following Fourier series was used to create the arterial inlet velocity function was the following:

$$f(t) = a_0 + a_1 cos(\omega t) + b_1 sin(\omega t) + ... + a_8 cos(8\omega t) + b_8 sin(8\omega t) \tag{1}$$

where $\omega = 7.257$ Hz and coefficients were as listed in Table 2.

Table 1. Fourier series coefficients for the arterial inlet velocity function

**Coefficient**	**value (with 95% confidence intervals)**
a_0	0.7417 (0.7417, 0.7417)
a_1	0.09529 (0.09529, 0.09529)
b_1	0.2639 (0.2639, 0.2639)
a_2	-0.05064 (-0.05064, -0.05064)
b_2	0.2378 (0.2378, 0.2378)
a_3	-0.1683 (-0.1683, -0.1683)
b_3	0.06663 (0.06663, 0.06663)
a_4	-0.03762 (-0.03762, -0.03762)
b_4	-0.008308 (-0.008308, -0.008307)
a_5	-0.07485 (-0.07485, -0.07485)
b_5	0.01985 (0.01985, 0.01985)
a_6	-0.03864 (-0.03864, -0.03864)
b_6	-0.05328 (-0.05328, -0.05328)
a_7	0.01286 (0.01286, 0.01286)
b_7	-0.02408 (-0.02408, -0.02408)
a_8	0.00647 (0.00647, 0.00647)
b_8	-0.0007462 (-0.0007463, -0.000746)
